# Supplementary material for: A model-informed target product profile for population modification gene drives for malaria control
Source: PLOS Glob Public Health. 2025 Aug 6;5(8):e0005026. doi: 10.1371/journal.pgph.0005026 (PMC12327634; doi:10.1371/journal.pgph.0005026)
Supplement: S1 Table — (PDF) [file pgph.0005026.s001.pdf]

S1 Table. Parameter values describing mosquito bionomics, vector control and malaria epidemiology.

Symbol	Parameter	Value	Reference
**Mosquito bionomics**			
β	Egg production per adult female (per day)	21	[1]
T_E	Mean duration of egg stage (days)	3	[1]
T_L	Mean duration of larval stage (days)	7	[1]
T_P	Mean duration of pupal stage (days)	1	[1]
CV(T_E)	Coefficient of variation, egg stage	0.2	[2]
CV(T_L)	Coefficient of variation, larval stage	0.3	[2]
CV(T_P)	Coefficient of variation, pupal stage	0.2	[2]
K	Larval carrying capacity	Time-varying	[3]
μ	Adult mosquito mortality rate	Time-varying	[3]
f	Blood feeding rate	1/3	[4]
Q	Human blood index	0.9	[4]
**Vector control**			
θ_B	Bites taken on humans while they are in bed as a proportion of all bites taken on humans	0.89	[5,6]
θ_I	Bites taken on humans while they are indoors as a proportion of all bites taken on humans	0.97	[5,6]
r_{LLIN}	Probability of repeating a feeding attempt in the presence of long-lasting insecticide-treated nets	0.56	[5,6]
r_{IRS}	Probability of repeating a feeding attempt in the presence of indoor residual spraying	0.60	[5,6]
s_{LLIN}	Probability of feeding and surviving in the presence of long-lasting insecticide-treated nets	0.03	[5,6]
s_{IRS}	Probability of feeding and surviving in the presence of indoor residual spraying	0	[5,6]
**Malaria epidemiology**			
N_H	Human population size	1,000	

v	Proportion of severe malaria cases that result in death	0.215	[7]

References

1. White MT, Griffin JT, Churcher TS, Ferguson NM, Basáñez MG, Ghani AC (2011) Modelling the impact of vector control interventions on *Anopheles gambiae* population dynamics. Parasites & Vectors 4:153.
2. Bayoh MN, Lindsay SW (2003) Effect of temperature on the development of the aquatic stages of *Anopheles gambiae* sensu stricto (Diptera: Culicidae). Bull Entomol Res 93:375–81.
3. Winskill P (2023) umbrella: Rainfall & seasonality. R package version 0.3.1.
4. Smith DL, Ellis McKenzie F (2004) Statics and dynamics of malaria infection in *Anopheles mosquitoes*. Malar J 3:13.
5. Le Menach A, Takala S, McKenzie FE, Perisse A, Harris A, Flahault A, Smith DL (2007) An elaborated feeding cycle model for reductions in vectorial capacity of night-biting mosquitoes by insecticide-treated nets. Malar J 6:10.
6. Griffin JT, Hollingsworth TD, Okell LC, Churcher TS, White M, Hinsley W, Bousema T, Drakely CJ, Ferguson NM, Basáñez MG, Ghani AC (2010) Reducing *Plasmodium falciparum* Malaria Transmission in Africa: A Model-Based Evaluation of Intervention Strategies. PLoS Med 7: e1000324.
7. Griffin JT, Bhatt S, Sinka ME, Gething PW, Lynch M, Patouillard E, Shutes E, Newman RD, Alonso P, Cibulskis RE, Ghani AC (2016) Potential for reduction of burden and local elimination of malaria by reducing *Plasmodium falciparum* malaria transmission: A mathematical modelling study. Lancet Infectious Diseases 16: 465-472.
